# Supplementary material for: In Vitro and In Vivo Evaluation of Human Adenovirus Type 49 as a Vector for Therapeutic Applications
Source: Viruses. 2021 Jul 28;13(8):1483. doi: 10.3390/v13081483 (PMC8402785; doi:10.3390/v13081483)

Supplemental Figure 1 (S1): Luminex quantification of cytokine and chemokine levels

HAdV-C5 or HAdV-D49 (1×10^{11}vp) vectors encoding luciferase were administered by *intravenous* injection Cytokine and chemokine analysis of sera was performed using a mouse cytokine 20-plex luminex panel quantifying levels of basic fibroblast growth factor (bFGF), granulocyte macrophage colony-stimulating factor (GM-CSF), interferon-gamma (IFN-γ), interleukin (IL) 1α, IL-1ß, IL-2, IL-4, IL-5, IL-6, IL-10, IL-12(p40/p70), IL-13, IL-17, IFN-induced protein (IP10), keratinocyte-derived cytokine (KC), monocyte chemoattractant protein (MCP-1), monokine induced by gamma interferon (MIG), macrophage inflammatory protein-1alpha (MIP-1α), tumour necrosis factor-alpha (TNF-α) and vascular endothelial growth factor (VEGF). IL-10, IL-13 and KC values < 1 and are therefore not plotted. Data and statistics are summarised in Figure 2E.

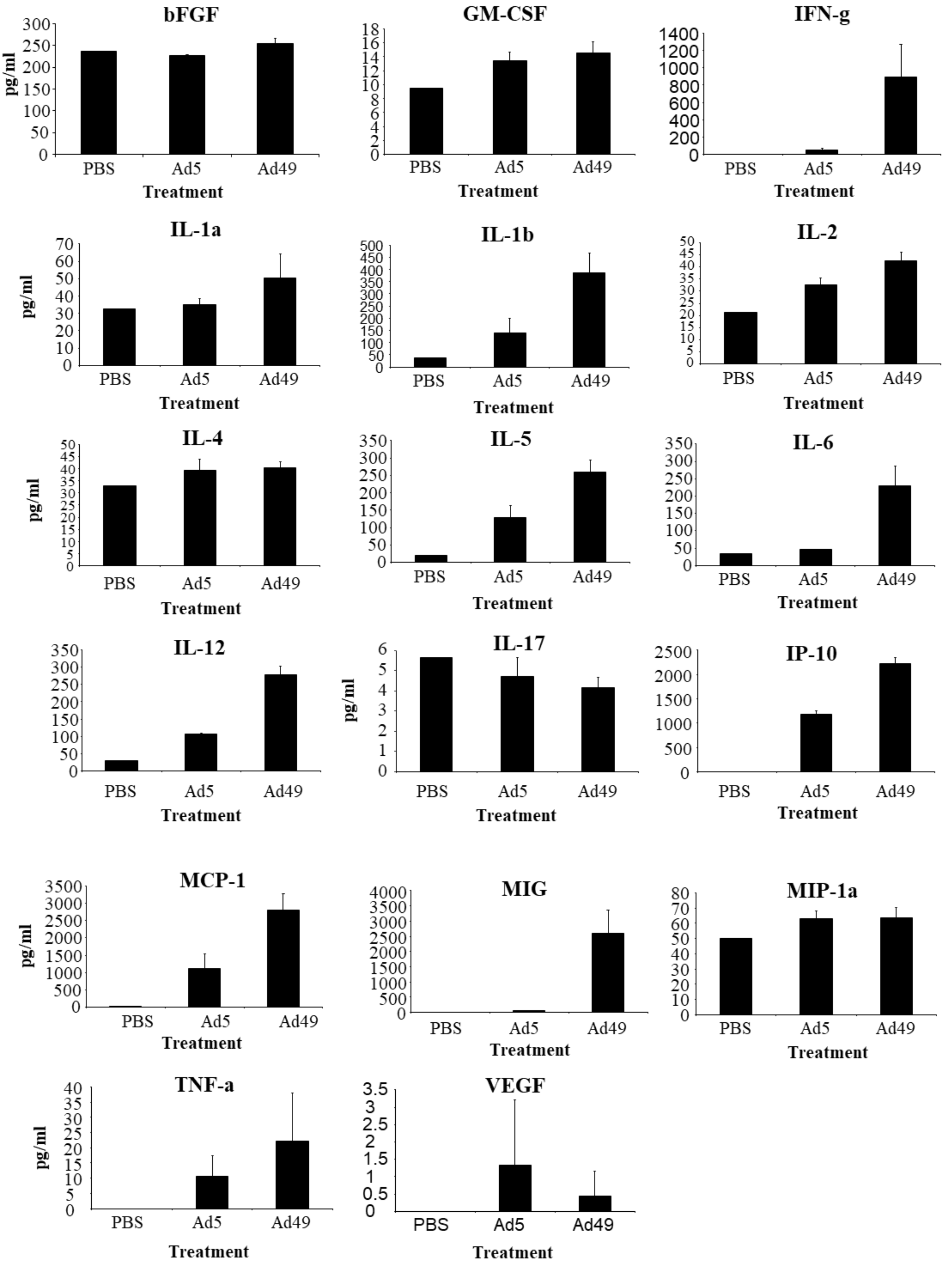

Supplement: Supplementary file 1 [file viruses-13-01483-s001.zip › viruses-1308346-supplementary.pdf]
